# Addressing System Challenges in Mental Health Services Research for Youth in the Child Welfare System: Insights From the Foster Care Family Navigator Trial

**DOI:** 10.1002/mhs2.70003

**Published:** 2025-01-28

**Authors:** Marina Tolou-Shams, Megan Ramaiya, Jannet L. Salas, Adrian Aguilera, Martha Shumway, Brian Borsari, Emily Dauria, Jill D. Berrick

**Affiliations:** 1 Department of Psychiatry and Behavioral Sciences, University of California, San Francisco, San Francisco, California, USA; 2 School of Social Welfare, University of California Berkeley, Berkeley, California, USA; 3 Center for Data to Discovery and Delivery Innovation (3DI), San Francisco Veteran Affairs Medical Center, San Francisco, California, USA; 4 Department of Behavioral and Community Health Sciences, Graduate School of Public Health, University of Pittsburgh, Pittsburgh, Pennsylvania, USA

**Keywords:** child-welfare-involved youth, disparities, foster care, health services, intervention, patient navigation, systemic barriers

## Abstract

Child welfare-involved (CWI) youth have high rates of unaddressed mental health needs, and system-level barriers (e.g., inadequate coordination between child welfare agencies and other systems designed to serve CWI youth) are one major reason why disparities in mental health services’ access continue to persist for CWI youth. This Research Note aims to inform the mental health services field about system-level challenges to conducting real-world, health services research with CWI youth and their families. We present challenges experienced in conducting our NIMH-funded Foster Care Family Navigator (FCFN) trial focused on development and preliminary testing of a clinic-embedded navigation intervention designed to improve detection of foster care youth services need, linkage to and engagement in community-based mental health services. Systems-level challenges that impacted proposed research trial design and data collection included: (1) Limited system staff time and compensation processes; (2) Staff training and knowledge; and (3) System disruptions. Health services research geared toward increasing access to mental health services to CWI youth must incorporate multiple complex design considerations prior to intervention development and delivery including longer (than 12 months) intervention development phases, detailed contingency plans for intervention delivery and integrated tailored, ongoing support and training for staff with existing clinic workflows. In this way, structural challenges to access to care that researchers are trying to ameliorate for CWI and other underserved, minoritized populations are not being inadvertently perpetuated through research study designs.

## 1 | Introduction

The number of youth in foster care in the United States is estimated at 368,500, with additional strains on systems and families noted due to the COVID-19 epidemic (US Department of Health and Human Services [USDHHS] 2024). These youth, referred to as child welfare involved (CWI), are considered a vulnerable population with high rates of untreated mental health needs (Bronsard et al. 2016; Taussig, Harpin, and Maguire 2014). Among CWI youth, 50% meet criteria for a current mental disorder, including anxiety and anxiety disorders (18%), depressive disorders (18%), attention-deficit/hyperactivity disorder (11%), and histories of suicidality (26.4%).

Despite the high documented need for mental health services, sustained gaps in identifying need and supporting services linkage and retention remain (Chuang and Wells 2010). For example, in one state with legally mandated mental health screening, only 28% of all youth were assessed for mental health needs within 2 months of case opening, 45% by 3 months, and 69% by 6 months. At the 1-year mark, 11% of youth had their case closed before ever receiving a mental health assessment. There are also notable disparities in mental health services access across racial and ethnic groups, with African American and Latiné youth less likely to receive mental health care compared with white youth (Garcia, Aisenberg, and Harachi 2012; Gudiño, Martinez, and Lau 2012). Poor coordination between child welfare and mental health service systems has been identified as a barrier to services access and utilization. Limited cross-talk between systems results in services redundancies and inefficiencies, inadequate services utilization, and confusion and burnout both for families as well as systems tasked with serving the mental health needs of CWI youth (Burns et al. 2004). The impact of undetected and poorly identified mental health needs among CWI youth are significant, and includes poor mental health outcomes following child welfare involvement, cross-over into the juvenile legal system and re-entry into the child welfare system, which exacerbates mental health, and perpetuates racial and ethnic disparities in mental health access (Bright and Jonson-Reid 2015). This Research Note is intended to inform the mental health services field about critical and timely structural challenges to address when conducting health services research studies with CWI youth populations and the mental health systems that serve them.

## 2 | Methods

We present insights from conducting an NIMH-funded “Foster Care Family Navigator” (FCFN) (R34MH119433; NCT04506437) study designed to develop and test preliminary efficacy of a family-based (youth/caregiver) mental health navigator intervention, delivered online and with navigation support through adjunctive digital health app. The FCFN intervention content was co-designed with and intended to be delivered by foster care mental health clinic staff. The aim of the intervention was to increase identification of mental health needs and enhance successful linkage from clinic assessment to community-based care for foster youth, ages 12–17 years (Tolou-Shams et al. 2023). The FCFN intervention spanned 6 months and was comprised of six, 60-min sessions delivered via telehealth using online intervention tools (i.e., interactive manualized content facilitated through PowerPoint slides) and tailored cross-system care coordination to support screening through linkage (Tolou-Shams et al. 2023). While not designed to be an implementation science trial, the intervention co-development process with frontline staff and child welfare systems leaders led to lessons learned about the “system’s readiness for change,” that we share as insights for researchers to consider when designing future implementation science trials with these systems and populations. This study was IRB-approved by the Principal Investigator’s university and the trial start date coincided with the onset of the COVID-19 pandemic in March 2020.

## 3 | Results

The research team encountered several structural challenges, including time availability limitations and challenges to compensation for the collaborating systems’ staff, uneven staff knowledge and training needs, and multiple system-level disruptions (e.g., high staff turn-over, staff shortages, workflow gaps) that impacted both the research study process and the ability to adequately detect and identify mental health needs of CWI youth. We describe these challenges and provides suggestions of ways that researchers can proactively attempt to address them, ideally at the initial study (and grant) conceptualization stage. In this way, we aspire to assist those who seek to develop and embed empirically supported approaches to reducing disparities in rates of mental health detection, identification, and screening among underserved, ethnoracial minoritized youth in the child welfare system.

### 3.1 | Partnering System Time and Compensation

When co-designing the FCFN intervention with systems and their staff, we found a 12-month intervention development phase (as proposed in the original grant) to be insufficient. Research grant timeline requirements that more closely match the workflow cadence of the system under study may lead to greater overall project success, particularly in under-resourced and over-burdened systems such as child welfare that lack sufficient, dedicated time to engage in additional, research activities. For example, an 18–24-month (start-to-finish) development phase could allow greater time for rapport building and thoughtful and thorough co-design of intervention delivery strategies and processes. Despite the increased timeframe and resources required to co-design interventions for ethnoracial minoritized, systems-impacted youth and families, the evidence is growing that co-design approaches in healthcare are critical for youth and family engagement, narrowing disparities and increasing equitable services’ access (Clark et al. 2021; Costanza-Chock 2020). System and staff involvement in research should also require ethical and equitable financial compensation for stakeholders involved in co-design (which are research activities above and beyond their daily mental health services provider tasks, such as intervention delivery), and a remuneration plan should be considered during the grant-writing process. If the system does not allow outside effort to be compensated (e.g., city- or state-contracted employees), then it is incumbent on the research team to meet early with administrators and other key stakeholders to develop a plan by which the system and staff can be remunerated for extensive time commitment. For example, in our work, we found that 90-min weekly stakeholder co-design meetings over 18 months could be continued as supervision meetings when the trial began, which leveraged clinic system existing workflows.

### 3.2 | Staff Training and Knowledge

The intervention co-design process does not equate to smooth and seamless implementation, given myriad structural challenges impacting even the most trained and knowledgeable staff. Providing training to staff, particularly non-specialists (i.e., those without formal training in mental health services delivery) is necessary and may be accelerated and enhanced through the intervention co-design process, but is not sufficient. Our original design of one-time trainings and weekly 60-min supervision felt insufficient to fully support navigators (who, in our study, were a combination of case manager, administrative staff, and unlicensed mental health clinicians), yet more tailored and ongoing support was not built into the original research project timeline or resources and was not possible due to competing obligations at the partnering clinic obligations. Ideally, 6-month booster trainings and weekly brief huddle supervision meetings in addition to the above would be negotiated with key partnering systems at grant conceptualization stage. At minimum, a more detailed assessment and discussion of existing clinic workflows ahead of a research grant submission may help to alleviate some of the challenges we confronted. The ideal situation with sufficient grant time and resources would be to consider a participant-observer method in which a member of the research team could be embedded in the partnering system to better understand the clinic workflows and day-to-day requirements of clinic staff that impact the very outcomes researchers are trying to study and change (e.g., mental health services screening, needs detection and linkage to care). Large-scale hybrid-type implementation science trials can also incorporate a structured system-level readiness for change assessment to best inform how to address system and staff challenges with intervention delivery.

### 3.3 | System Disruptions

Multiple studies have demonstrated that the COVID-19 global pandemic in 2020 drastically widened pre-existing disparities in health services access for publicly insured, ethnoracial minoritized populations (Dailey, Talleyrand, and Goodman 2024; Berger and Miller 2021). For example, our partnering clinic suffered numerous staffing gaps and leadership turn-over during the 3-year project period with many positions remaining unfilled for 6–12 months; this directly impacted our plan to solely rely on clinic staff as navigators for intervention delivery. Research teams must prepare contingency plans for intervention delivery in advance (particularly if in the midst of an efficacy trial when these structural challenges with partnering systems arise) that maintain methodological rigor and also equity in access for enrolled youth and families. We adopted a strategy of classifying all navigators as non-specialists (i.e., unlicensed clinicians) and one licensed clinical navigator as supervisor (who had to be county clinic staff), which allowed for study navigators who were part of the university research team to deliver the intervention due to high staffing turnover.

## 4 | Conclusions

In sum, health services research geared toward increasing access to mental health services to CWI youth requires complex early detection, screening, and navigation study designs that incorporate multiple systems considerations ahead of research data collection. The collateral consequences of not thoughtfully addressing some of these real-world barriers in designing and conducting such health services research studies are significant and can include youth crossing over into the juvenile legal system (i.e., getting into legal trouble for behaviors that represent unaddressed mental health needs while in foster care). As the juvenile legal system is often operating as the de facto behavioral health system of care, social injustice and inequities are perpetuated when need is not detected adequately, consistently and systematically, thus limiting access to appropriate and empirically informed interventions that promote optimal child outcomes. The path to transformation of access to mental health services for adolescents in the child welfare system requires detailed consideration of these complex real-world research challenges, advocacy for enhanced necessary resources to conduct this research rigorously and incorporation of flexible and creative, research methodology.

## Data Availability

The authors have nothing to report.
